# Analyzing the association between heat and utilization of inpatient care: evidence from Dresden University Hospital (Germany)

**DOI:** 10.1186/s12889-025-24804-8

**Published:** 2025-10-02

**Authors:** J. Thiel, A. Staudt, S. Grummt, M. Sedlmayr, E. Henke, J. Weidner

**Affiliations:** 1 Institute for Medical Informatics and Biometry,, Faculty of Medicine and University Hospital Carl Gustav Carus, TUD Dresden University of Technology,, Fetscherstraße 74, Dresden, 01307 Germany; 2 Institute and Policlinic of Occupational and Social Medicine,, Faculty of Medicine and University Hospital Carl Gustav Carus, TUD Dresden University of Technology, Fetscherstraße 74, Dresden, 01307 Germany

**Keywords:** Heat impact on health, Heat-related hospitalization, Heatwaves, Hospitalization, Climate change

## Abstract

**Background:**

As a result of climate change, temperatures and the burdens on human health are rising. This study investigates the relationship between acute heat and hospitalizations for specific diseases at the University Hospital Carl Gustav Carus in Dresden, Germany.

**Methods:**

A statistical analysis was conducted using clinical data from 4,363 patients treated between January 1, 2018, and December 31, 2023. Data included demographic information and heat-related diagnoses categorized by codes from the International Classification of Diseases. Climatic data was sourced from the Dresden-Klotzsche weather station, focusing on days with air temperatures exceeding 23 °C (warm days) and 30 °C (heat days). Correlation analyses were performed to assess the relationship between heat events and hospital admissions on these days.

**Results:**

We found a positive correlation between hospital admissions and heat at temperatures ≥ 23 °C for all included diseases combined (*p* < 0.05). A positive correlation was observed between temperatures ≥ 23 °C and hospitalizations for the diseases dehydration (E86), stroke (I63) and chronic obstructive pulmonary disease (J44) (all *p* < 0.05). At temperatures of ≥ 30 °C, no statistically significant correlation was identified. We discovered that men, young children and older people were particularly affected by heat-related diseases.

**Conclusions:**

The findings based on routine data from one German university hospital indicate that moderate heat can impact hospitalizations for certain diseases, at temperatures above 30 °C, no evidence for a statistically significant association was found, possibly due to a lack of statistical power or behavioural adaptations. This underlines the need for low-threshold preventive measures in response to the health risks associated with rising temperatures.

**Supplementary Information:**

The online version contains supplementary material available at 10.1186/s12889-025-24804-8.

## Background

Globally, further temperature increases are projected as a consequence of climate change, with both the intensity and duration of heatwaves expected to grow [1]. The impacts of climate change are diverse and with varying effects – for example heat waves, droughts, floods, storms, and other extreme weather events [2]. The World Health Organization assumes that such events will continue to increase as a result of climate change and expects up to 250,000 deaths and an additional burden on the healthcare system between 2030 and 2050 as a result [1]. In Germany, rising air temperatures and the resulting diseases pose a significant challenge [1]. This is particularly relevant as the optimum temperature for humans is around 17–19 °C and a deviation from this can lead to an increase in mortality and morbidity [3, 4]. It is not clearly defined exactly which temperature limits are to be understood as heat and when they are relevant for health risks. This is also evident in the World Health Organizations (WHO) recommendations for preventing heat-related diseases, where various thresholds between 25 °C and 35 °C are discussed, depending on individual and other climatic factors [5]. The Robert-Koch-Institute, the institute responsible for the management of health risks in Germany [6], employs a similar approach, setting thresholds starting at around 20 °C and above for estimating heat-related health impacts depending on the region, age group and observation period [7].

The strain on human health is manifold and manifests itself not only in disorders of the fluid balance or heat strokes [8], but also, for example, in the form of cardiovascular events like heart attacks or heart failure [8], or respiratory diseases like asthma or chronic obstructive pulmonary disease (COPD) [9]. The health system is also increasingly burdened by the health consequences of heat [10]. Back in 2017, the German Federal Ministry for the Environment, Nature Conservation, Building and Nuclear Safety provided recommendations for developing heat action plans to protect human health. Accordingly, heat action plans should be created and evaluated at the state and local authority levels [11, 12]. An example of this is the heat action plan already implemented in the United Kingdom, which activates different warning levels and corresponding measures in summer depending on the climatic conditions [13]. More specific predictions are also possible, as shown, for example, by Nishimura et al. They used retrospective treatment data and weather data to predict future patient numbers as a consequence of heat for the city of Nagoya in Japan [14]. In this context, digital tools can play a useful role, although they have not yet been widely used in Germany, leaving potential untapped [15]. To advance the digitalization of the healthcare system in Germany, the Federal Ministry of Education and Research supports for example the Medical Informatics Initiative and six Digital Health Progress Hubs. The Medical Informatics Hub in Saxony (MiHUBx) is one of these six Digital Health Progress Hubs aiming to develop a tool for predicting hospital utilization during acute heat events [16]. To develop such a predictive model, specific relationships between acute heat and individual diseases in saxony must first be investigated and understood. To our knowledge, there is insufficient empirical evidence regarding the associations shown between individual diseases and the utilization of the healthcare system, particularly of hospitals in Germany or even Saxony or Dresden. In order to be able to take into account the specific factors and correlations of the region in the prediction model, these must first be examined.

The primary aim of this study is to investigate the relationship between acute heat and the utilization of inpatient care for patients with certain diseases using clinical data from the University Hospital Carl Gustav Carus Dresden in the federal state of Saxony in Germany. This analysis should serve as a foundation for optimizing preventive measures, particularly for predicting resource utilization in Saxony.

## Methods

### Data sources and data preparation

The clinical data was provided by the University Hospital Carl Gustav Carus Dresden via its Data Integration Center (DIC). The selection of diseases and other characteristics analyzed was based on a prior literature review about heat-diseases [17], a mapping based on this review [18] as well as on the study protocol and ethics application developed for MiHUBx. This work was approved by the ethics committee at TU Dresden (SR-EK-479112023/2024).

The analyses included patients of all ages who had their current residence in Dresden during the study period from January 1, 2018 to December 1, 2023 and who were hospitalized as inpatients (including emergency department) in at the University Hospital Carl Gustav Carus Dresden for diseases related to heat. Hospitalizations were defined as the total number of admitted cases in a certain period of time. The diagnoses were presented using the International Classification of Diseases, Tenth Revision, German Modification (ICD-10-GM) [19]. Specifically, cases with the following ICD-10-GM codes were retrieved: E86 (Volume depletion), I20 (Angina pectoris), I21 (Acute myocardial infarction), I63 (Cerebral infarction), I64 (Stroke, not specified as haemorrhage or infarction), J44 (Other chronic obstructive pulmonary disease), L55 (Sunburn), T67 (Effects of heat and light), as well as any applicable subgroups of these codes. For the statistical analyses, the ICD-10-GM codes were not examined at the level of individual subgroups (e.g. J44.0, J44.1, J44.8, J44.9). Instead, the analysis was carried out at the level of the hierarchy group (e.g. J44), where subgroups like J44.1, J44.8. etc. where included, in order to enable a superordinate view of the disease cases. In addition, further treatment data was obtained, treatment-related data were obtained, including pseudonymized patient ID, visit start and end dates. Also, demographic data such as patient age, grouped in 5-year increments (0–4 years, 5–9 years, etc.), gender, and place of residence (based on the first three digits of the postal code) were retrieved.

In addition, climatic data from the regional climate information system (ReKIS) [20] at the Dresden-Klotzsche weather station was used. To describe climatic conditions, daily mean and maximum air temperatures (T) were selected, as these are frequently cited in the literature as indicators of heat stress [17]. The mean temperature provides insight into the general daily stress, while the maximum temperature specifically reflects peak stress for the human body [21, 22].

Two different temperature thresholds will be used in our study. First of all, a daily maximum temperature of 23 °C or higher is used to define a “warm day”. This value falls within a range commonly used in the literature, as described in the previous paragraph, and was previously applied in a study by Hüsing et al. [23] in Germany. In addition, the typical definition of a heat day in Germany of 30 °C and higher [24] was selected as second temperature threshold.

**Fig. 1:**
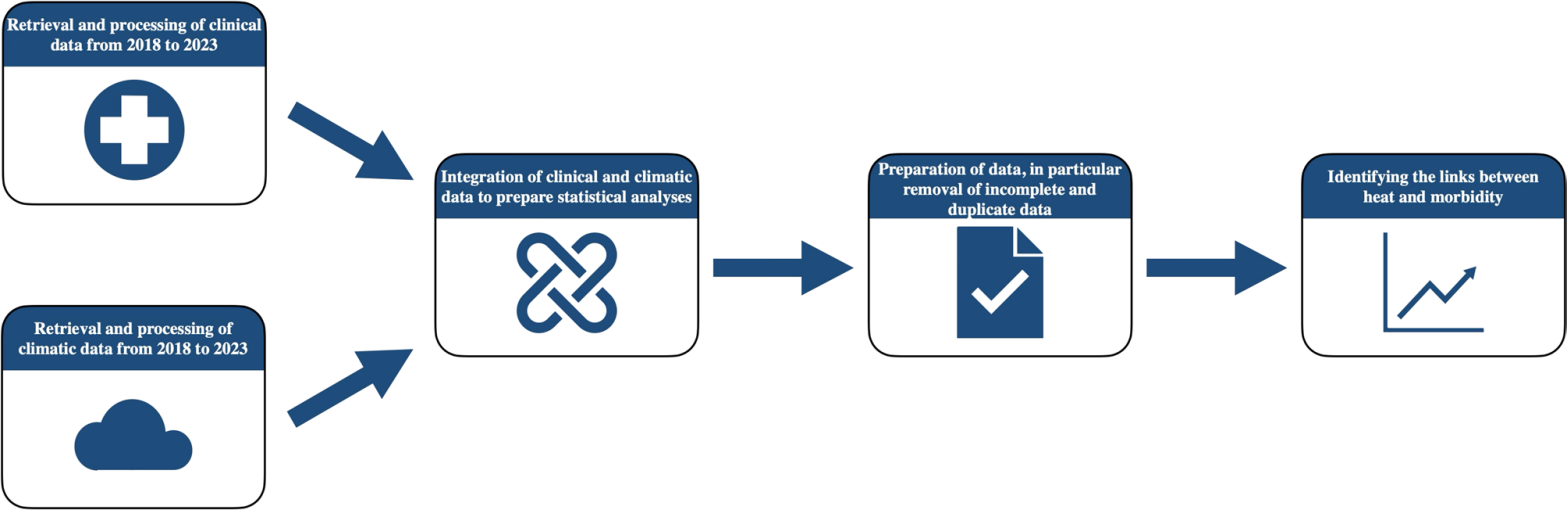
Retrieval and preparation for analyzing the study data

In preparation for the statistical analysis, each treatment case with a temperature of 23 °C or more on the day of admission was assigned the corresponding maximum temperature. Incomplete data records and duplicate data records were removed (Fig. 1).

### Statistical analysis

The statistical analysis for the period between January 1, 2018, and December 31, 2023 was conducted using RStudio (R version 4.4.1) [25]. First, the clinical data was analyzed. This involved examining the included patients, the total number of cases, as well as the annual counts of cases. The distribution of gender and age groups was also considered, along with a combination of age and gender distribution and the absolute frequencies per age group. In addition, the length of stay (based on visit start and end dates) was analyzed for each of the hierarchy group assigned to ICD-10-GM.

Furthermore, the climatic data was examined. This included determining the number of warm days, defined as days with a maximum temperature of 23 °C or higher and the heat days with 30 °C or higher, over the entire observation period as well as annually. The development of annual and monthly average temperatures over the observation period was also examined.

To analyze correlations, the relationship between selected diseases based on the ICD-10-GM hierarchy group and climatic heat was examined using a Pearson correlation analysis (r), followed by a two-sided t-test for statistical significance (α = 0.05) and the calculation of the 95% confidence interval (CI).

For the significant results the (relative) rate ratio (RRR) and a Chi-square test (α = 0.05) for statistical significance were calculated between men and women and between different age groups in order to determine if there are any risk groups. The age groups were divided into infants (0–4 years), children and adolescents (5–19 years), adults (20–59) and seniors (60+). In addition, the 95% CI was calculated for the RRR in each case.

A lag time of 0 days was used for the correlation analysis, meaning that hospital admissions on the same day as the heat event were examined, as the investigation by Peng et al. indicated that this yields the strongest relationship [26]. For the analysis, daily temperature values were rounded to whole numbers to ensure consistent categorization. To minimize distortions caused by lower numbers of very hot days, the average patient count for each day with a specific temperature was calculated. This ensured a consistent evaluation of patient numbers in relation to temperature and minimized the potential impact of rare, extremely high temperatures (i.e., days > 35 °C). To ensure an adequate sample size and statistical power, only diseases that accounted for at least 5% of the total cases in the dataset were included in the correlation analysis.

## Results

### Description of heat-related diseases

**Fig. 2:**
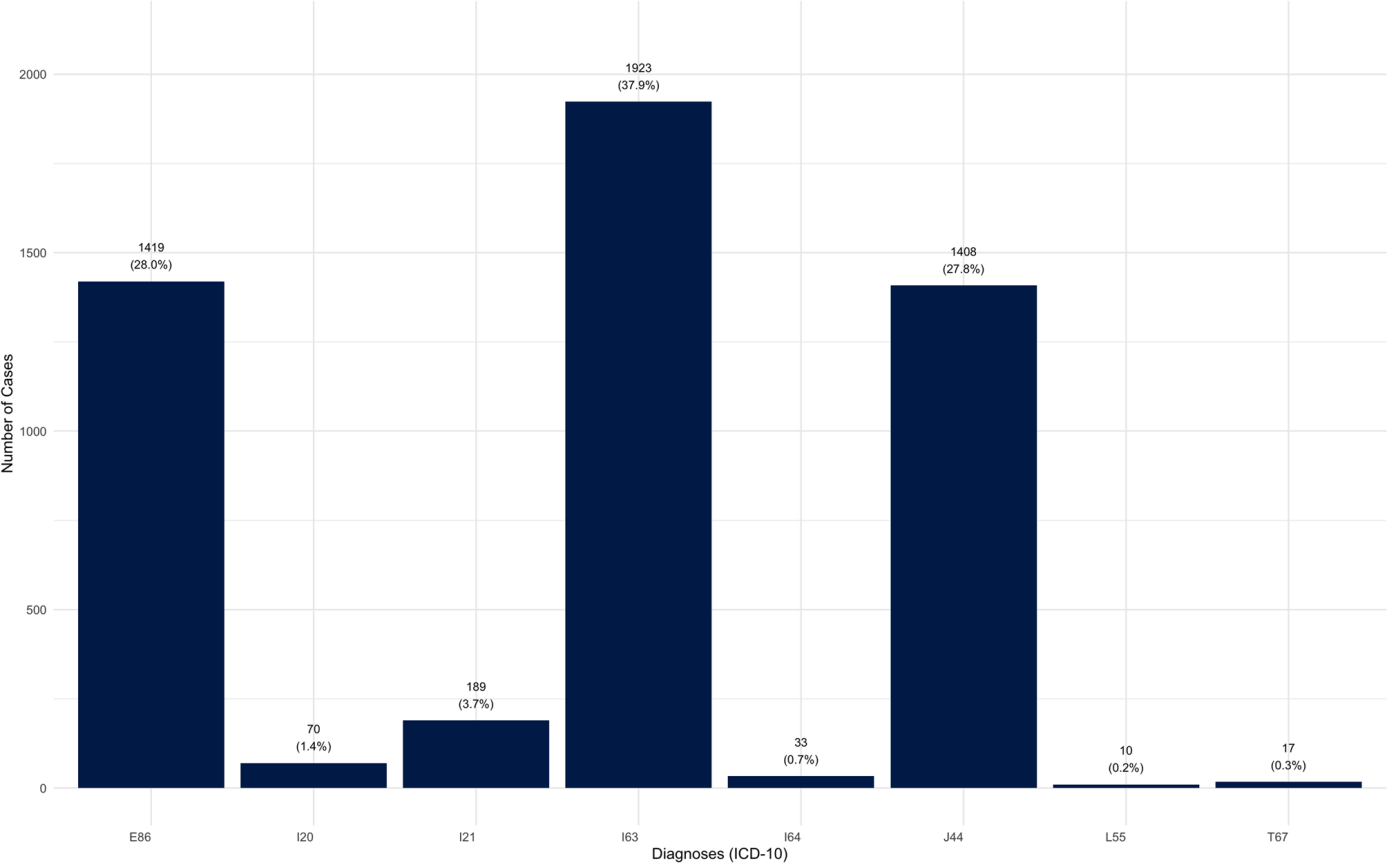
Frequency of the specified diagnoses in the observation period

During the observation period from 2018 to 2023, diagnoses corresponding to the ICD-10-GM codes E86, I20, I21, I63, I64, J44, L55, and T67 with overall 5,069 treatment cases were reported by the DIC Dresden. The majority of diagnoses were strokes (I63), cases of volume depletion (E86) and COPD (J44). These three main groups accounted for 93.7% of all treatment cases related to the included diagnoses (Fig. 2).

### Description of the study population

A total of 4,363 patients with at least one of the specified hierarchy diagnoses were treated at the University Hospital Dresden during the study period from 2018 to 2023. In total, 5,069 treatment cases with one of the above mentioned diseases were recorded. The annual treatment numbers ranged from 751 to 983 cases per year, with a noticeable decline in case numbers observed since 2020 (Table 1).

**Table 1:** Number of cases per year

Year	Cases
2018	983
2019	939
2020	844
2021	755
2022	797
2023	751
∑	5,069

**Fig. 3:**
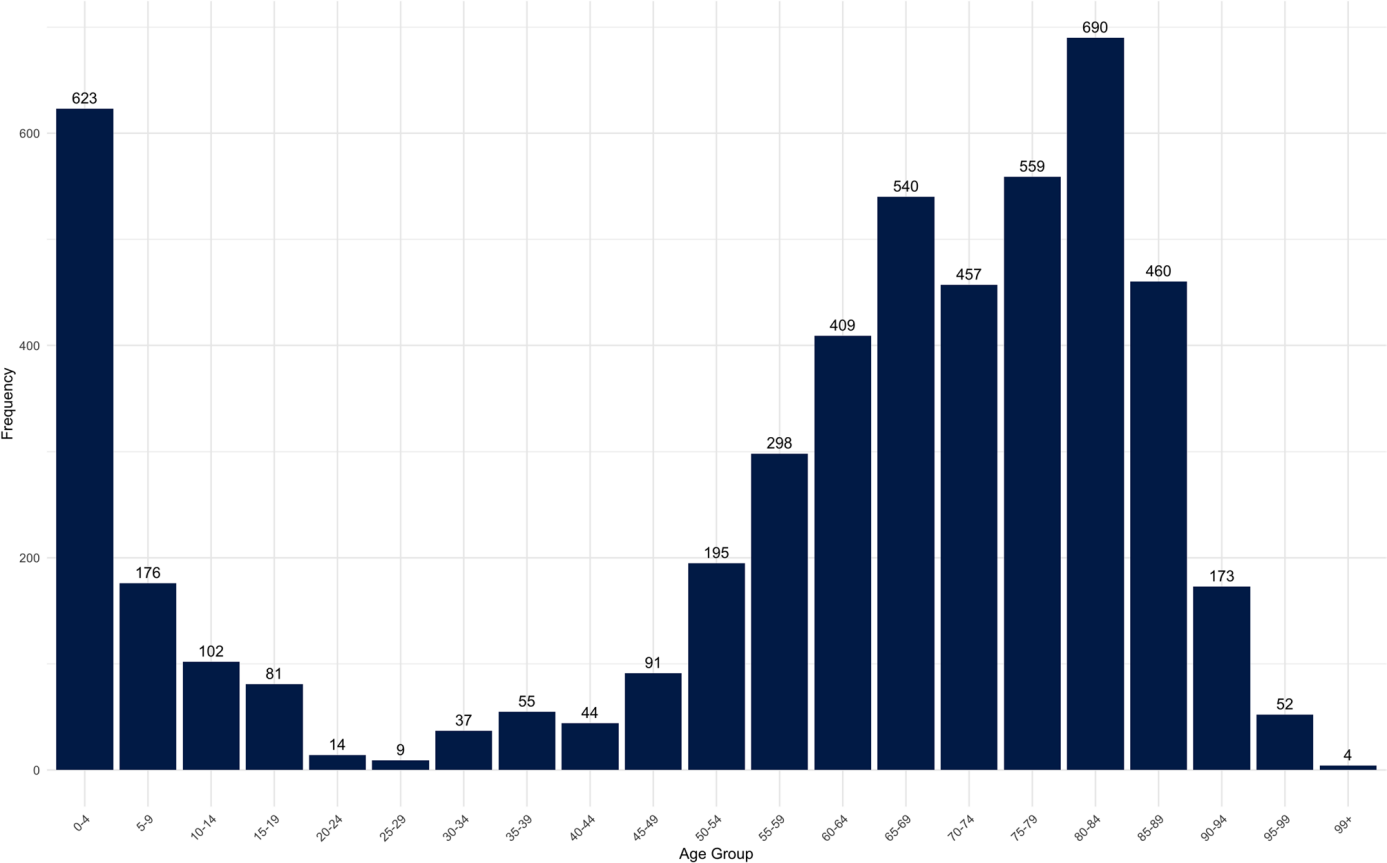
Age distribution of the study population

**Fig. 4:**
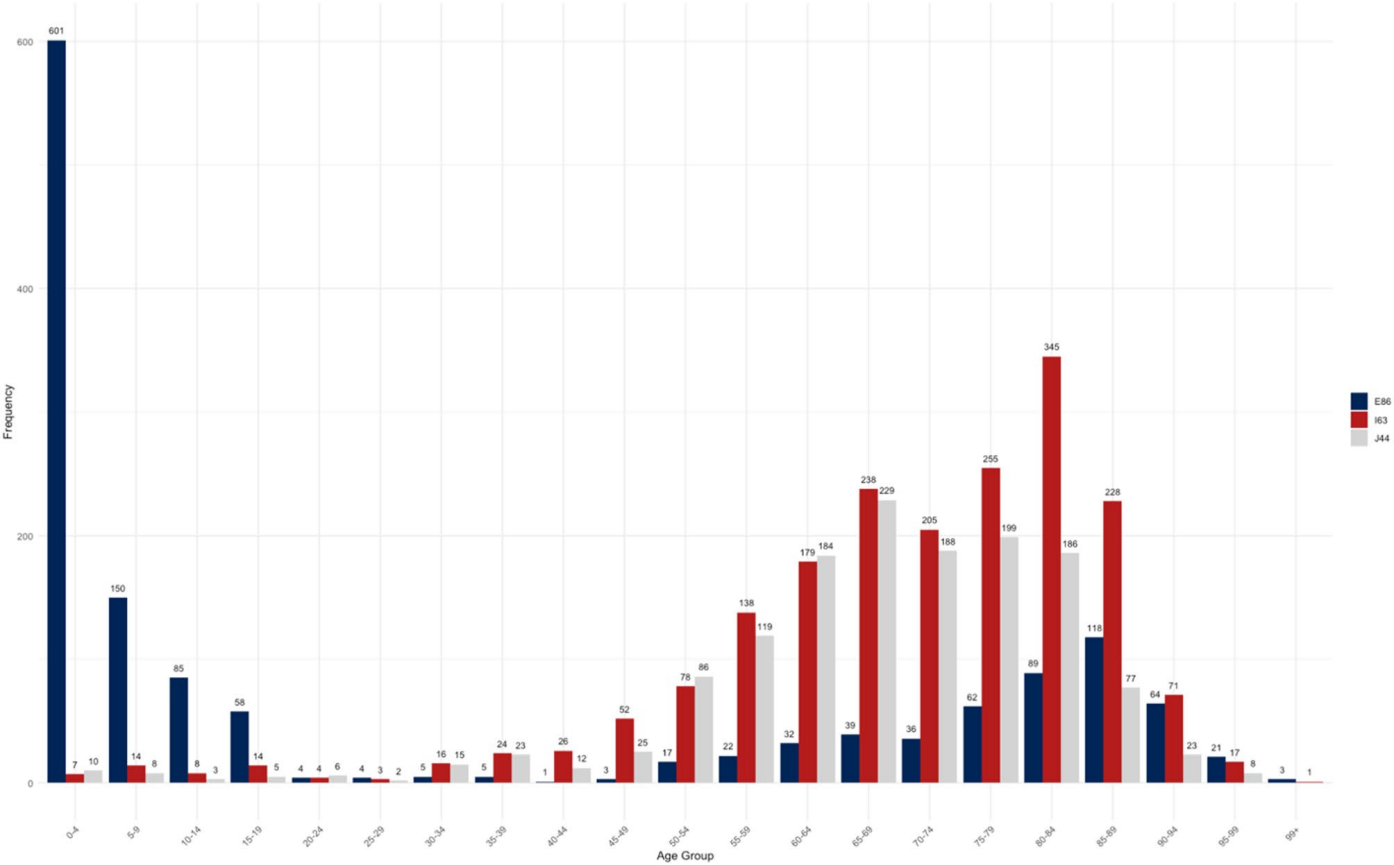
Age distribution of the individual diagnoses

The average length of stay for all diagnoses was 7.6 days with a standard deviation of 12.3 days. A detailed presentation of the length of stay of all diagnoses together and the three diagnoses that make up each at least 5% of the data set, is shown in Table 2. The examination of age distribution (Figure 3) shows that especially children aged 0 to 4 years and older adults between 80 and 84 years are affected of the analyzed diseases. It has also been shown that younger patients were treated for the diagnosis E86, while older patients were more frequently admitted for I63 and J44 (Figure 4). It was also identified that the proportion of men in the study population (54.6%) is statistically significantly (p < 0.05) larger than the proportion of women (45.4%) (Appendix 1).

**Table 2:** Number of cases and average length of stay in days for the analyzed diagnoses

Diagnosis code	Number of Cases	Mean (Standard deviation)
All diagnoses	5,069	7.6 (12.3)
E86 – Volume depletion	1,419	5.5 (8.6)
I63 – Cerebral infarction	1,923	6.7 (8.9)
J44 – COPD	1,408	11.0 (17.5)

### Description of climatic factors

**Fig. 5:**
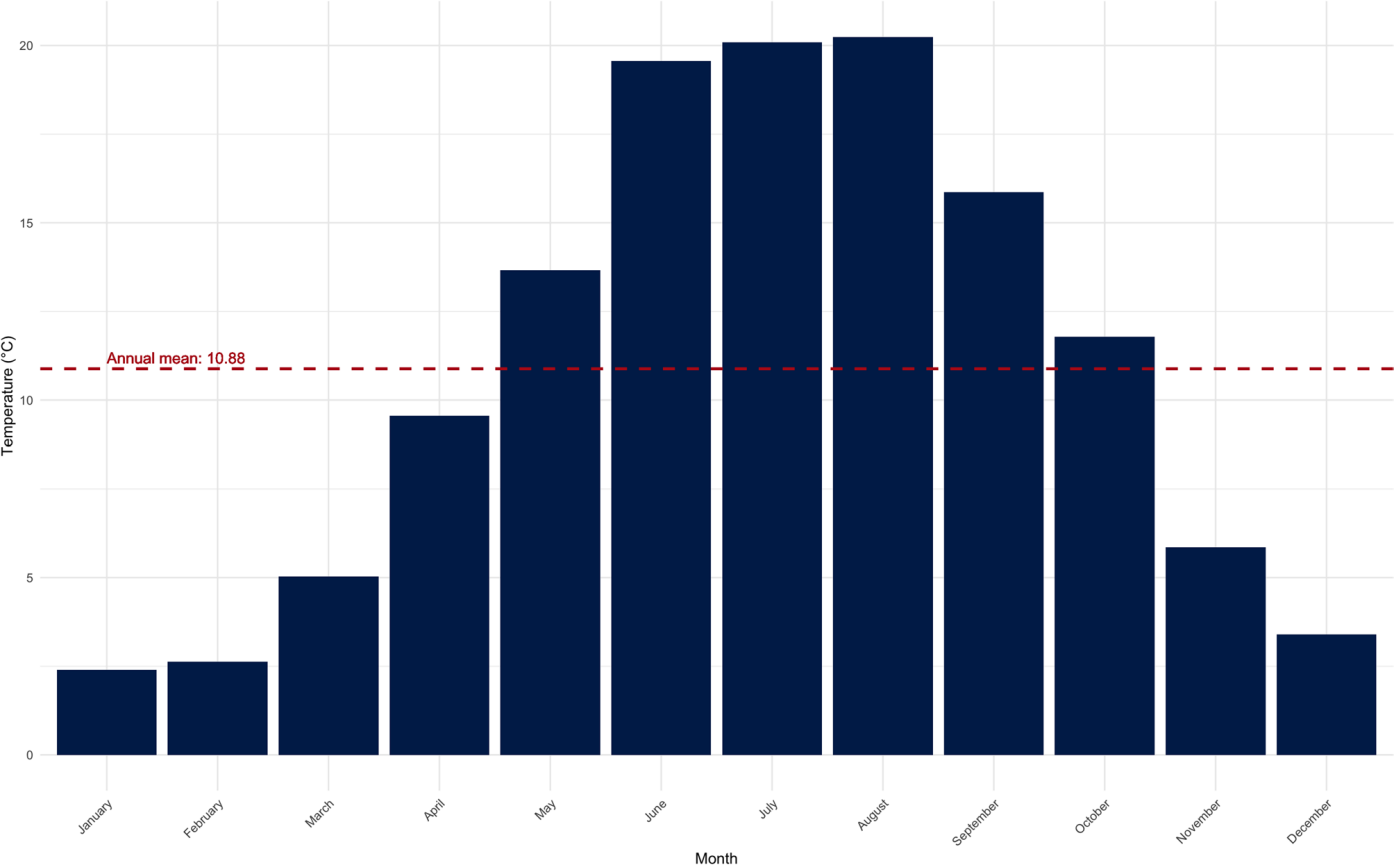
Monthly average temperatures in the observation period

During the observation period from 2018 to 2023, a total of 504 days (23.0% of all days in that period) with a maximum temperature of 23 °C or more and 100 days (6.4% of all days in that period) with at least 30 °C were identified. The annual number of warm and heat days can be found in Table 3. The overall mean temperature for the observation period was recorded at 10.88 °C, with the period from May to October showing above-average temperatures, while the period from November to April exhibited below-average temperatures (Fig. 5).

**Table 3:** Number of warm and heat days per year

Year	Warm days (T ≥ 23 °C)	Heat days (T ≥ 30 °C)
2018	108	28
2019	80	25
2020	82	14
2021	71	4
2022	75	17
2023	88	12
Σ	504	100

### Correlation Analysis

**Fig. 6:**
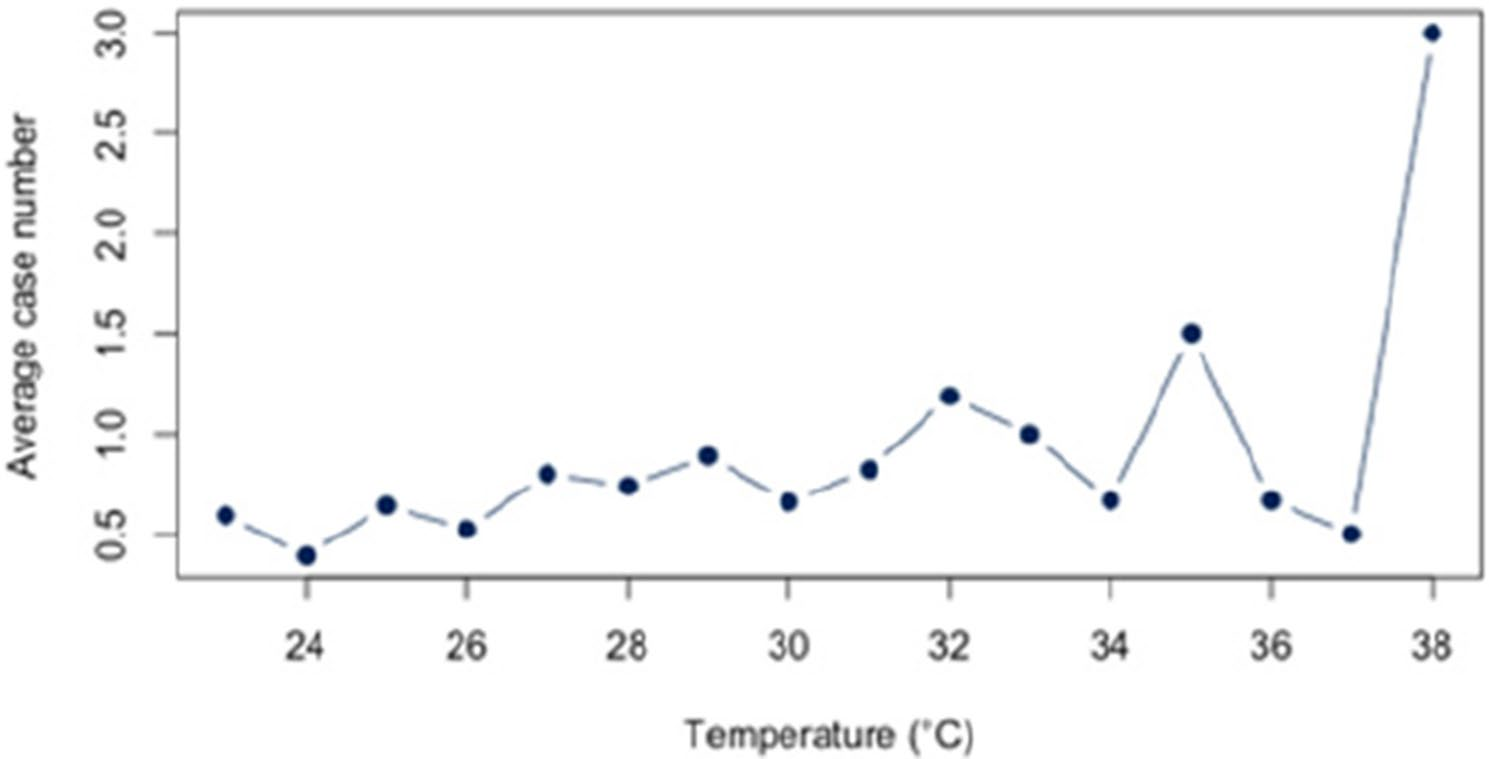
Link between E86 and Heat (T ≥ 23 °C)

**Fig. 7:**
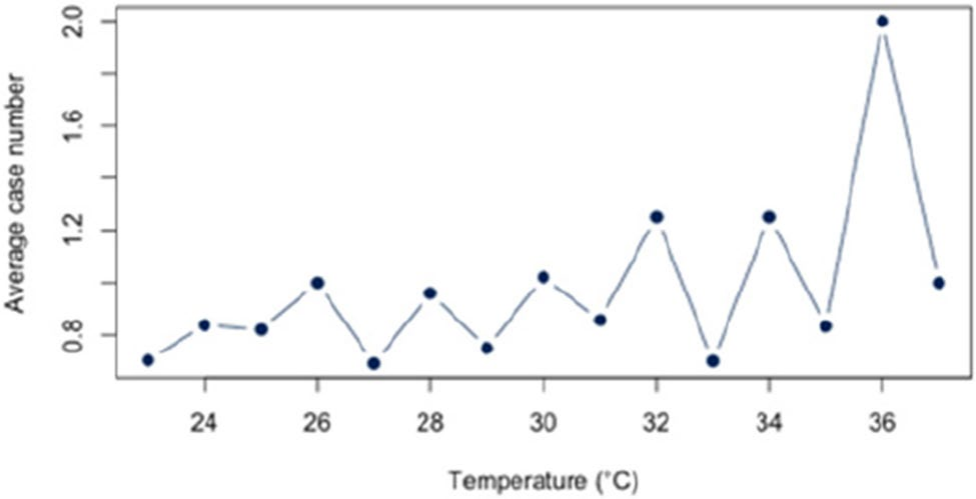
Link between I63 and Heat (T ≥ 23 °C)

**Fig. 8:**
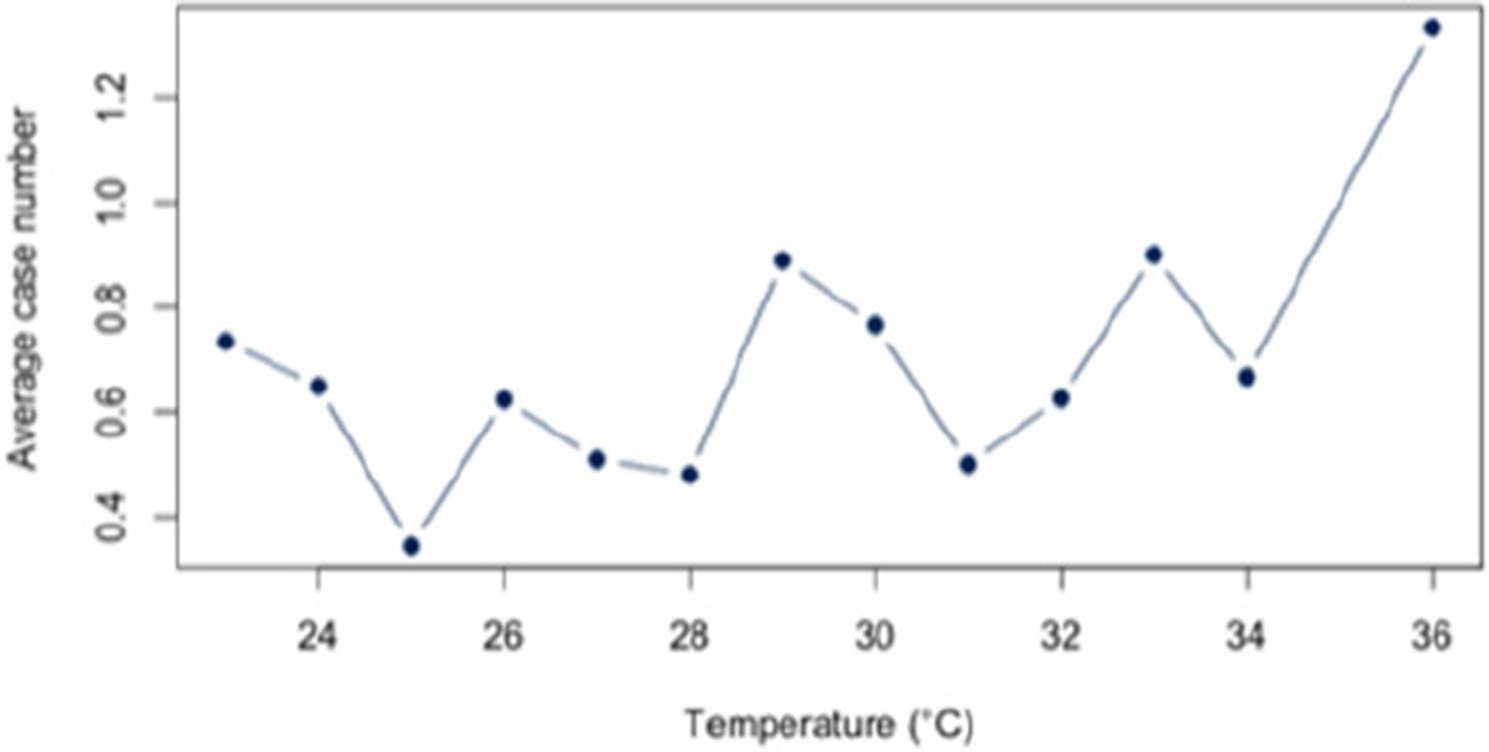
Link between J44 and Heat (T ≥ 23 °C)

First, we analyzed the relationship between heat and hospital utilization of selected diseases at temperatures of 23 °C and above (Appendix 2). For all diagnoses together, a correlation of *r* = 0.63 (*p* < 0.05) was found. The correlation analysis for each disease revealed significant relationships between heat and the number of cases for each of the three diagnoses (E86, I63, J44) that account for at least 5% of the dataset. For the diagnosis E86, a positive correlation of approximately *r* = 0.54 (*p* < 0.05) was observed. Also, a positive and statistic significant correlation was demonstrated for the diagnosis I63 (*r* = 0.52; *p* < 0.5) and J44 (*r* = 0.56;*p* < 0.05). An overview about the correlations at 23 °C or more can be found in Figs. 6, 7 and 8. The correlations and p-values are shown in Table 4.

**Table 4:** Results of the statistical correlation analysis

Diagnosis code	Pearson correlation coefficient (95% CI; *p*-Value) for warm days (T ≥ 23 °C)	Pearson correlation coefficient (95% CI; *p*-Value) for heat days (T ≥ 30 °C)
All Diagnosis	0.63 (95% CI [0.20;0.86]; *p* = 0.008)	0.14 (95% CI [−0.58;0.74]; *p* = 0.127)
E86	0.54 (95% CI [0.06;0.82]; *p* = 0.031)	0.46 (95% CI [−0.29;0.86]; *p* = 0.209)
I63	0.52 (95% CI [0.01;0.82]; *p* = 0.048)	0.35 (95% CI [−0.47;0.85]; *p* = 0.398)
J44	0.56 (95% CI [0.02;0.85]; *p* = 0.044)	0.74 (95% CI [−0.19;0.97]; *p* = 0.096)

**Fig. 9:**
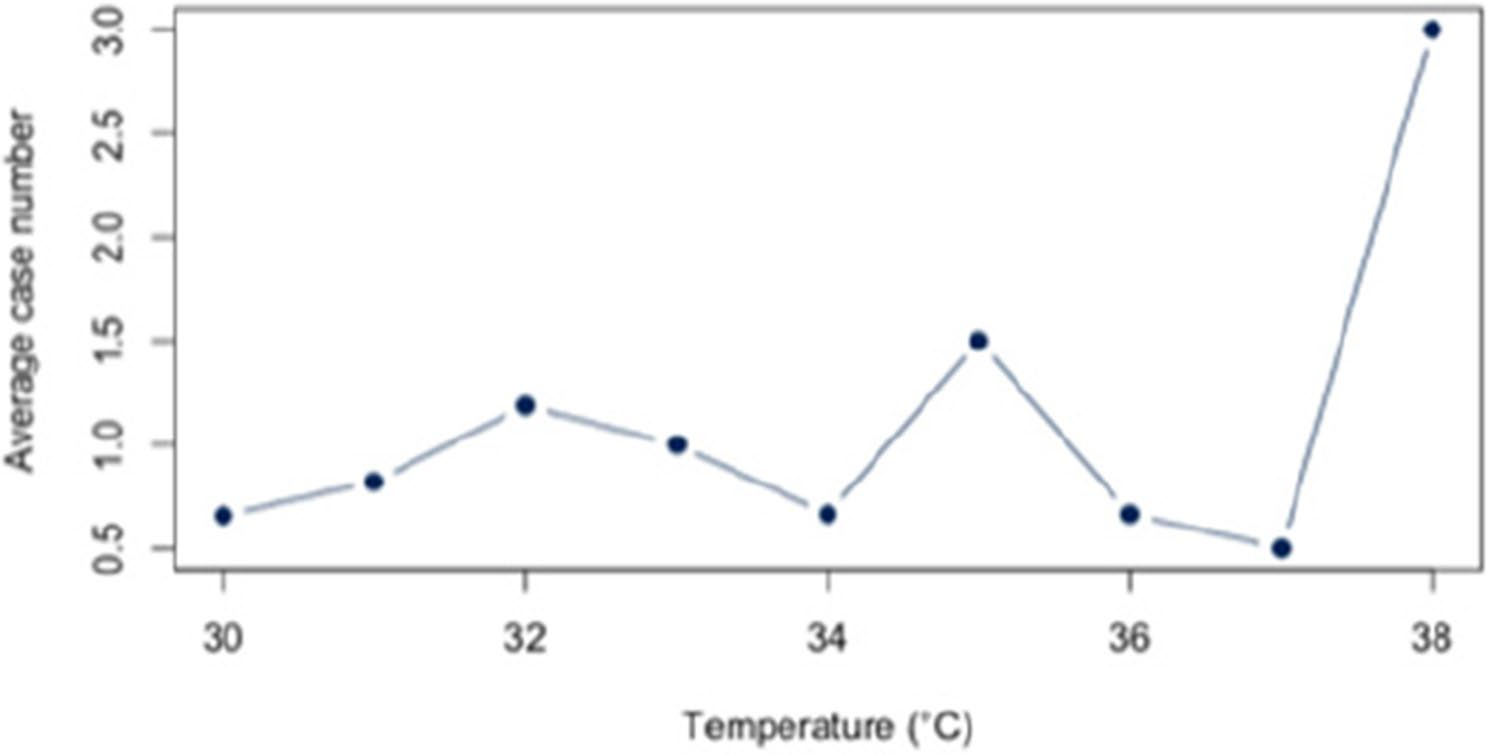
Link between E86 and Heat (T ≥ 30 °C)

**Fig. 10:**
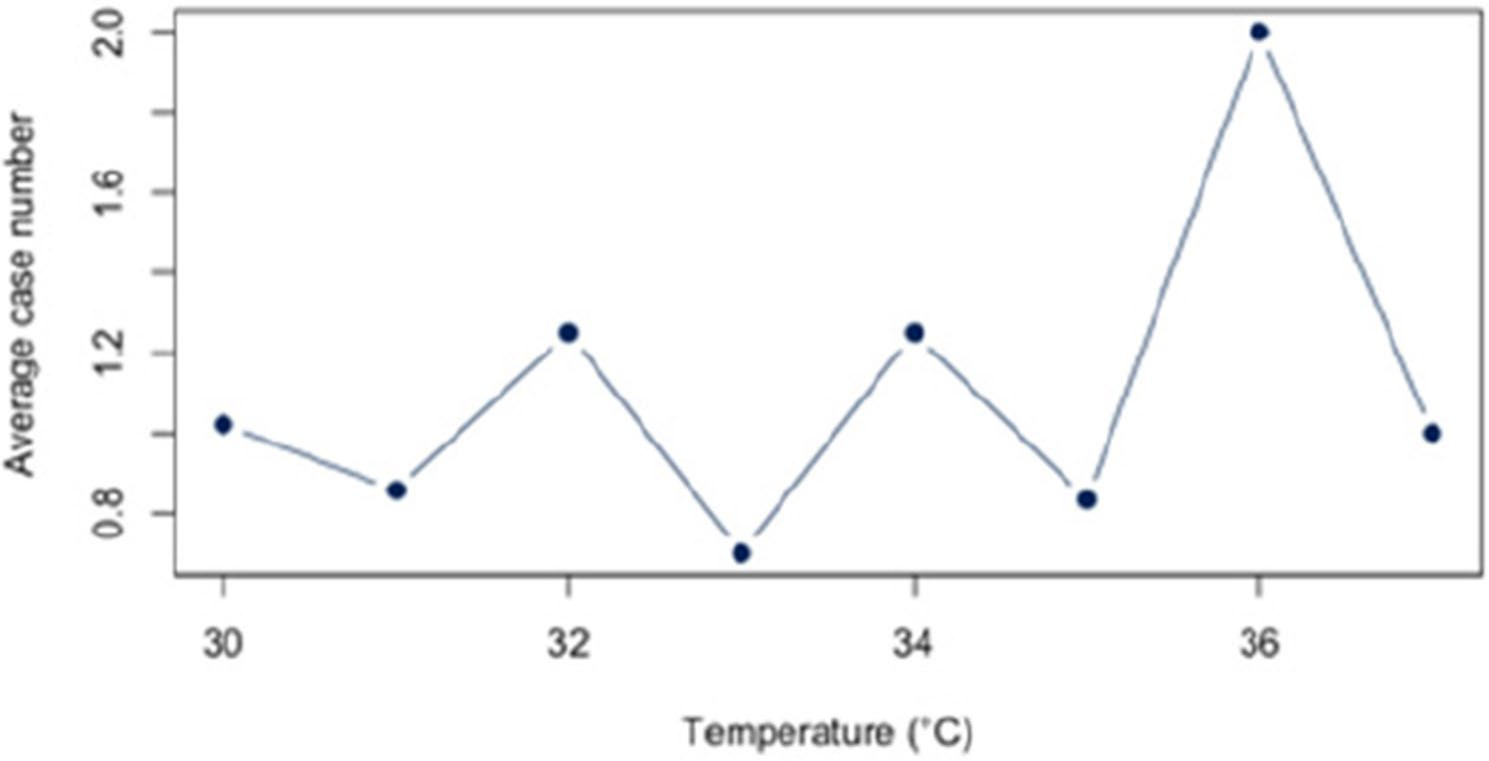
Link between I63 and Heat (T ≥ 30 °C)

**Fig. 11:**
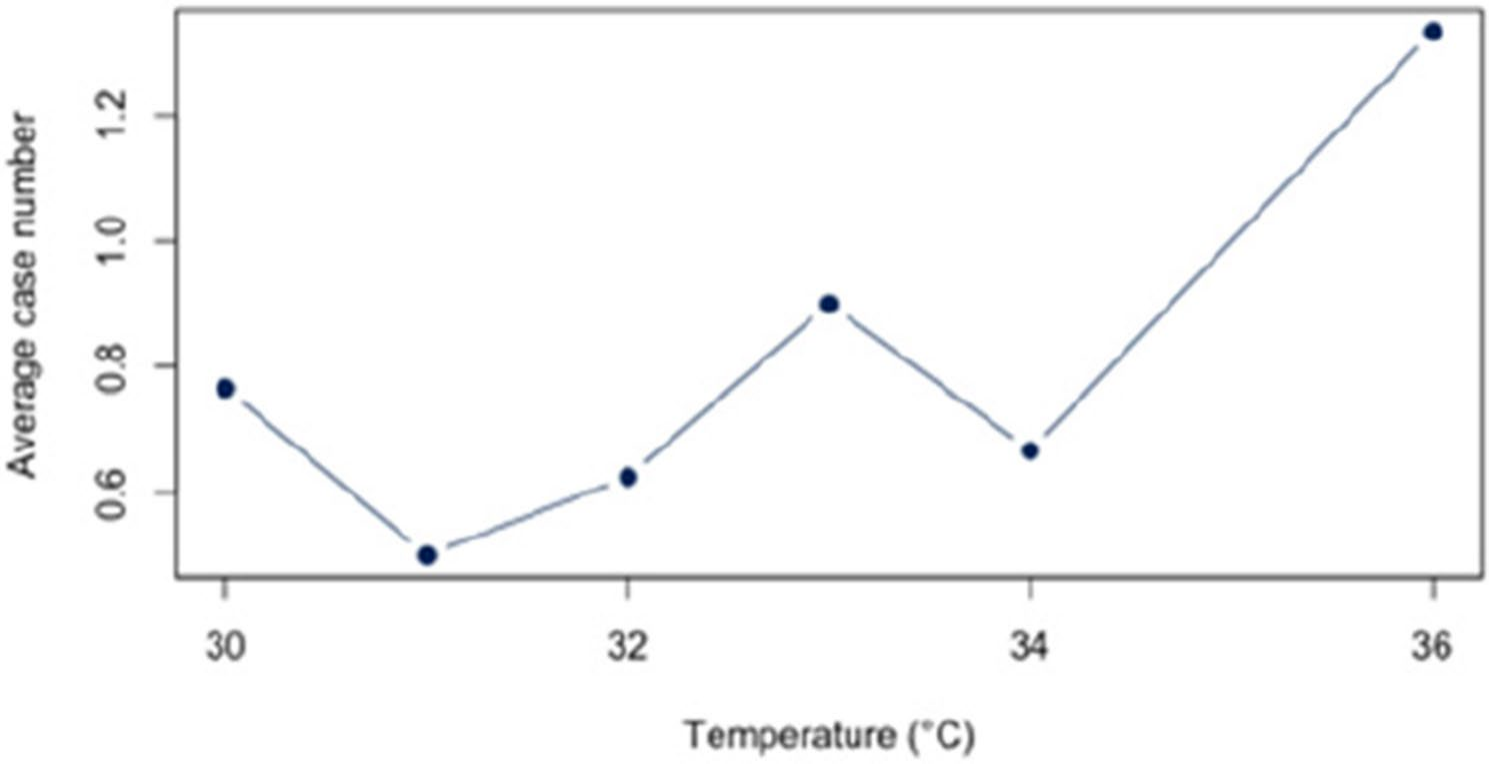
Link between J44 and Heat (T ≥ 30 °C)

At 30 °C or more for all diagnoses combined (Appendix 3), no statistically significant correlation (*r* = 0.14; *p* > 0.05). For the examination of individual diseases, the strongest correlation was found for J44 (*r* = 0.74; *p* > 0.05). For E86 (*r* = 0.46; *p* > 0.05) and I63 (*r* = 0.34; *p* > 0.05), low to moderate correlations were found. Figures 9, 10 and 11 provide detailed illustrations. The detailed overview about the correlation and the p-value is shown in Table 4. Due to the lack of significance, no further analyses were carried out for this threshold value of ≥ 30 °C.

The calculation of the rate ratios for gender showed that at ≥ 23 °C, men (RRR = 1.26; *p* < 0.05) have a statistically significant higher risk than women for all diagnoses combined. For the diagnosis E86, men have a statistically non-significant lower risk than women (RRR = 0.98; *p* > 0.05), while for I63 (RRR = 1.08; *p* > 0.05) and J44 (RRR = 1.61; *p* < 0.05) the male gender has an increased risk compared to women (Appendix 4). The analysis between the different age groups and diagnoses showed that, for all diagnoses together, children under 5 years have a higher risk compared to adolescents (5–19 years). The comparison between children under the age of 5 and senior citizens also showed an increased risk for senior citizens. For the diagnosis E86, the risk for children under 5 was significantly higher than for all other age groups. For diagnoses I63 and J44, the risk for seniors was higher than for younger age groups. A detailed presentation can be found in Appendix 5.

## Discussion

The primary objective of this study, to investigate the relationship between heat and hospital occupancy, was achieved by combining routine hospitalization and climate data over a period of five years. With our study, we were able to demonstrate a correlation between the hospital utilization for all included heat-related diagnoses combined, as well as between the specific diagnoses E86, I63 and J44 and exposure to climatic heat for warm days from 23 °C onwards, suggesting higher number on admission for the health system on warm summer days. The analysis of relationships of hot days starting at 30 °C could not demonstrate any statistically significant correlations with heat-related hospitalizations. However, it should be noted that treatments in the context of heat, especially above 30 °C, account for the minority of cases.

### Statistical methods

For the correlation analysis, the Pearson coefficient was used, thus exclusively examining linear relationships. Considering linear associations when estimating heat-related morbidity is common, as exemplified by the study of Patenge et al. in Munich (Germany), which also employed Pearson correlation to estimate heat-related hospitalizations [27]. Furthermore, a study comparing statistical approaches analyzing general, not only heat-related healthcare utilization found that linear models are at least equivalent to, and sometimes better than, more complex models such as e.g. random forest algorithms [28].

Nevertheless, healthcare utilization is influenced by additional factors, such as seasonal and calendar effects like weekdays or holidays [29], further environmental factors including air pressure, humidity, UV radiation, as well as patient-specific risk factors such as pre-existing conditions, age, or sex [17]. The relative risk ratios observed in this study may suggest the influence of these factors. However, as no confounding variables were explicitly considered in the correlation analysis, this interpretation remains limited and also limits the robustness of the conclusions. In contrast to other international studies that adjusted for a range of environmental and demographic variables [30].

Future research should involve further analyses using alternative study designs, such as cohort studies [31] or the use of a kind of case-cross-over design in which heat periods can be compared with control periods, as well as more advanced statistical methods. Non-linear causal relationships should be taken into account as well, e.g. by using Spearman’s correlation or more complex regression analyses such as fractional polynomials. In particular, multivariate methods should be pursued in order to take other influencing variables into account and identify confounding variables.

#### Literature context

Due to the heterogeneity of the diagnoses and climatic influences examined, it is challenging to contextualize these findings within the existing literature. Nonetheless, the results are in line with national and international research showing clear associations between healthcare utilization and thermal stress [17]. The statistical analysis by Peng et al., which examined hospital admissions in Shanghai (China) at different temperature thresholds, also found an increase in admissions in moderate heat. In addition, the data set does not include all cardiology cases, as the cardiology ward at Dresden University Hospital is an independent legal entity and the data is therefore not currently recorded together [26]. Statistical analysis of publicly available data from East Sussex (United Kingdom) from Jackson and Noushad on hospital admissions and subsequent mortality showed an increase in mortality in summer [32]. who were able to prove an increased mortality rate in the heat in their time series analysis with death figures in the Netherlands [4]. Similar effects for the diagnoses examined (E86, I63, J44) have been demonstrated by various research teams and are described as follows. For instance, Anderson et al. found a positive correlation between hospital admissions and temperature for COPD in the many different counties in the USA [33]. Green et al. similarly demonstrated heat association for strokes in different California counties (USA) [34, 35]. In Germany, analyses by Augustin et al. based on health insurance claims data also showed a link between heat and treatment numbers for conditions such as volume depletion [36]. It should be noted that our analyses do not allow direct conclusions about the exact influence of heat on the pathogenesis of volume depletion, stroke, and COPD. However, our work, like the other studies listed above, suggests that heat can significantly increase hospital admissions. The analyses also suggest that men, young children and older adults may have a higher risk of hospitalization due to heat-related diseases and should therefore be given special attention. The findings are consistent with the existing literature, which has identified similar risk groups for the diseases mentioned [17]. It should be emphasized that the individual diseases occur with different frequencies in the respective risk groups. Children in particular are primarily affected by E86, while older people are more likely to be hospitalized with J44 or I63. It should be noted that the risk groups were quantified using risk ratios. This measure exclusively reflects the ratio of individuals with the disease between the groups and does not account for the proportion of individuals without the disease. Due to the lack of data on non-diseased people, other measures such as the odds ratio that include comparison groups could not be used [37]. Nevertheless, the age- and gender-specific risk groups are not atypical. The literature shows that men are generally admitted to hospital more frequently [38]. It is also known from the literature that dehydration is particularly significant in young children [39]. Similar observations can be made in older patients with regard to COPD [40] and strokes [41]. Although this indicates that risk groups are not exclusive to heat-related illnesses, it underscores the significance of risk group analyses.

#### Regional relevance

To our knowledge, this is the first study to demonstrate effects specific to the Dresden area. Significant effects could only be demonstrated for temperatures for the threshold value of 23 °C. The reasons for the lack of evidence of significant effects at the threshold value of 30 °C are unclear. For example, Peng et al. describe physiological limits, possible adaptation effects with regard to behavior and causes based on statistical methods [26]. Similar reasons could also be relevant in our study. Possible causes for the lack of effects at ≥ 30 °C could be the low number of very hot days as well as adaptation effects and protective measures. It is conceivable that due to the low number of days with 30 °C or more, the statistical power decreases and the detection of actual effects becomes more difficult. However, the observed differences between the thresholds of 23 °C and 30 °C could also be due to successfully implemented adaptation strategies in Dresden, as expressly recommended in the healthcare sector [42]. The city of Dresden has issued specific health guidance for heat, explicitly addressing health impacts at temperatures of 30 °C or higher [43]. It is possible that adherence to this guidance from 30 °C onwards could reduce the disease burden. The findings suggest that preventive measures, such as early warning systems or heat action plans, should also take milder heat periods into account and not only temperatures of 30 °C or more. Moreover, the rather low daily case numbers found for certain temperatures do not indicate a need for predictive models just for inpatient care. Further studies are needed to identify other health impacts of heat on the healthcare system and to be able to develop appropriate measures. Also, further studies should be carried out for more in-depth investigation, ideally involving a larger number of cases. In addition, separate studies on possible adaptation effects could be carried out.

### Strengths and limitations

To our knowledge, this analysis is the first to quantify the correlation between heat and hospital admissions in Saxony at a municipal level. The analyses include a multi-year study period with over 5,000 included cases. The study only included data from one hospital. Due to the separation of inpatient and outpatient treatment in Germany, it is not possible to make any assertions about the outpatient sector. In addition, the data set does not include all cardiology cases, as the cardiology ward at Dresden University Hospital is an independent legal entity and the data is therefore not currently recorded together. In general, it should be noted that the study only provides an overview of a few selected diseases. However, it should be noted that the selection was based on a previously conducted meta-review at a high methodological level [17]. It should also be mention that the included sunburn is not caused directly by heat, but by UV radiation and is therefore not a heat disease in the strict sense. Nevertheless, sunburn was taken into account in the analyses, as UV radiation and heat tend to occur in summer periods [44] and are closely linked [45, 46].

Furthermore, only data from one weather station were considered, meaning that localized heat islands may not have been captured, potentially leading to additional biases. The district is also located on the outskirts of the city in the north and is characterized primarily by the airport and as an industrial location [47]. This may have led to an underestimation of the heat load compared to residential areas in the center of the city. However, there was no alternative to using this weather station, as it was the only one with comprehensive and complete data for the period under investigation. It should also be noted that the selection of the threshold value of 23 °C can be limiting and did not follow a fixed definition. It should be noted that there are currently no standardized threshold values and that the selected value is based on another study in Germany [23] and is within the usual definition starting at arround 20 °C [6]. However, the utilization of standardized diagnoses in the form of ICD codes can facilitate the reproducibility of the study at other sites and reduce the existing limitations.

## Conclusion

The results of the study show an association between daily temperatures above 23 °C and hospitalizations, indicating a potential strain on the healthcare system in the Dresden region related to the diseases under investigation. Even moderate heat events lead to a statistically significant increase in hospital admissions. No evidence for an association between daily temperatures above 30 °C and hospitalizations were found, possibly due to too few heat days in the study sample. The analysis further reveals that specific risk groups are disproportionately affected during periods of moderate heat. In particular, men, young children, and the elderly are shown to be especially vulnerable. These findings underscore the importance of targeted public health interventions, such as the implementation of early heat warning systems, focused awareness campaigns for at-risk populations, and the adaptation of healthcare infrastructure to withstand increasing climatic stress.

The study highlights the urgent need for comprehensive action to combat climate change, not only to reduce future warming and the frequency of extreme weather events but also to mitigate their growing impact on health systems. The healthcare sector must prepare for climate-related challenges, but this adaptation must go hand in hand with ambitious climate protection measures to limit further health risks. The dual approach of mitigation and adaptation is essential to safeguard both population health and the resilience of medical institutions.

To better assess the burden posed by heat-related health risks and to develop more effective mitigation strategies, further research is essential. Future studies should ideally employ more robust statistical methods and incorporate a wider range of climatic and patient-specific variables. Additionally, expanding the geographical scope of the research beyond Dresden would help improve the generalizability of the findings and facilitate a deeper understanding of regional differences in vulnerability and health system resilience.

## Supplementary Information

Supplementary Material 1

## Data Availability

The climatic data that support the findings of this study are available from the Regional Climate Information Service of the Technical University Dresden can be viewed publicly on their website. The medical data are held by Data Integration Center of the University Hospital Carl Gustav Carus Dresden at the Faculty of Medicine of the Technical University of Dresden. Due to privacy and data protection regulations, the data are not publicly available and can only be accessed upon request directly from the Data Integration Center of the University Hospital Carl Gustav Carus Dresden at the Faculty of Medicine of the Technical University of Dresden. Likewise, the R code used for data analysis is currently not publicly accessible because it cannot yet be ensured that all components fully comply with data protection regulations, that would be necessary if they were published. However, upon formal request and approval, the corresponding analysis code may be made available together with the data.
