# Supplementary material for: Analyzing the association between heat and utilization of inpatient care: evidence from Dresden University Hospital (Germany)
Source: BMC Public Health. 2025 Oct 2;25:3293. doi: 10.1186/s12889-025-24804-8 (PMC12492696; doi:10.1186/s12889-025-24804-8)
Supplement: Supplementary file 1 — Supplementary Material 1 [file 12889_2025_24804_MOESM1_ESM.docx]

**Appendix**

Appendix 1: Age and gender distribution

**
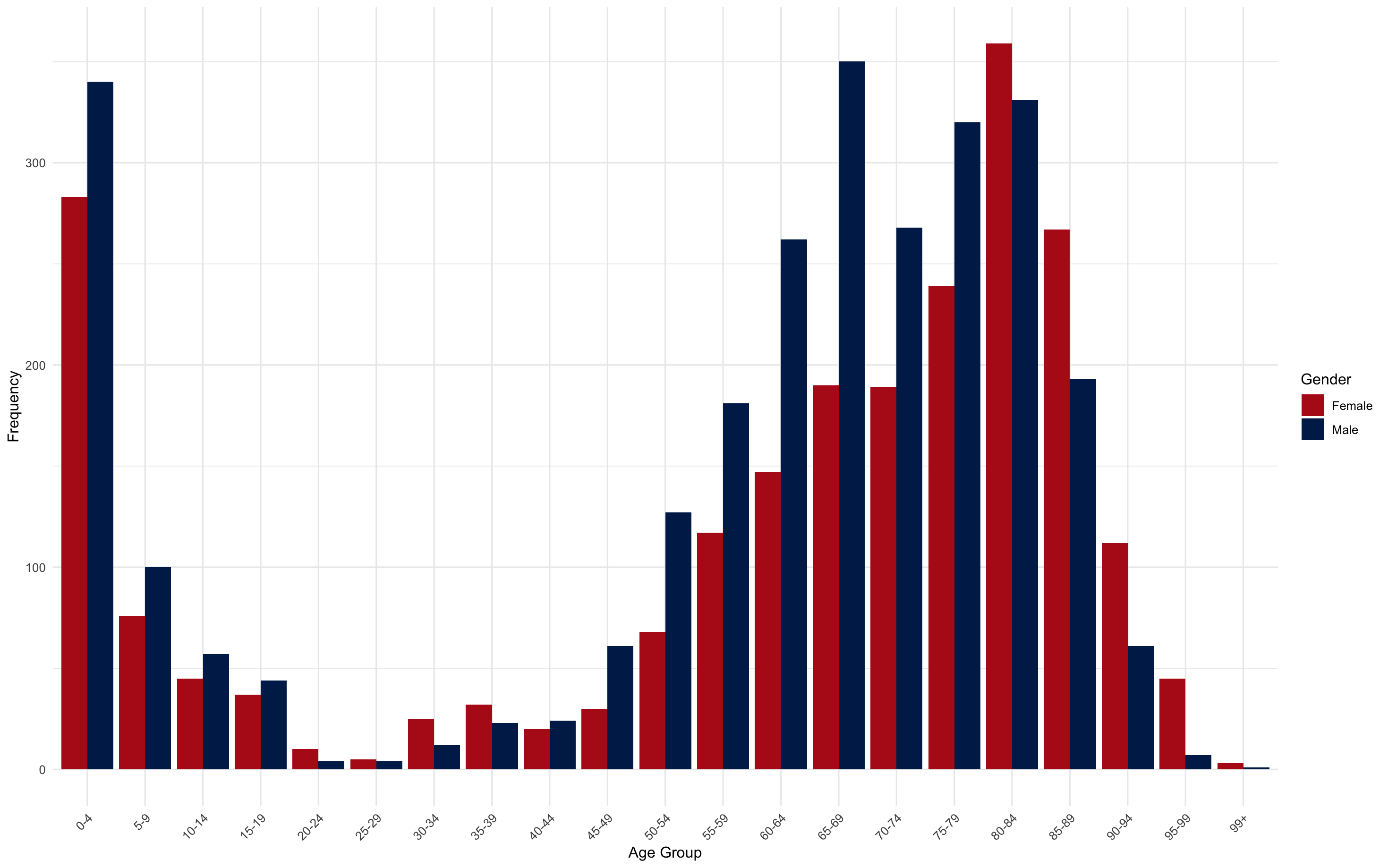
**

Appendix 2: Link between all diagnoses and temperature for ≥ 23°

**
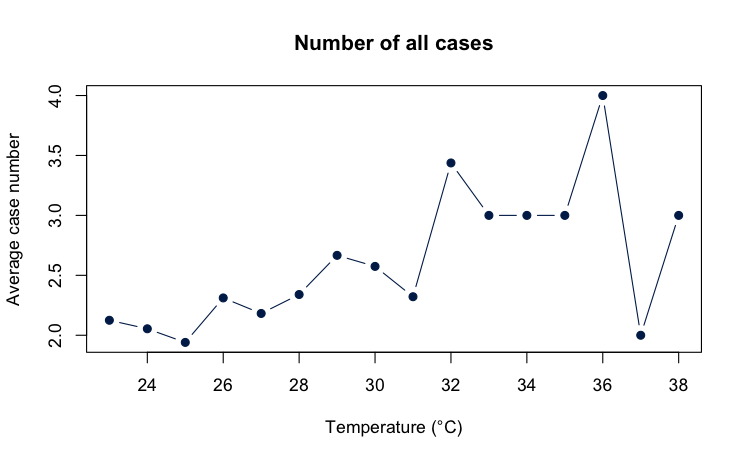
**

Appendix 3: Link between all diagnoses and temperature for ≥ 30°


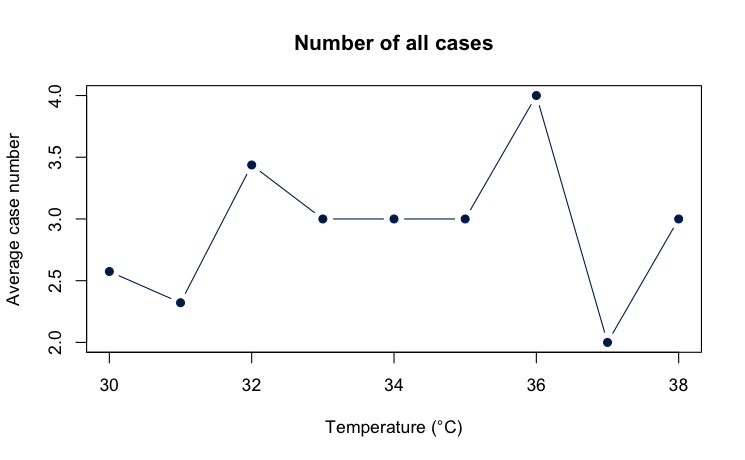


Appendix 4: Rate Ratio of men compared to women for ≥ 23°C

| **Diagnosis code** | **Relative Rate Ratio (95% CI; p-value)** |
| --- | --- |
| All Diagnosis | 1.26 (95% CI [1.14; 1.27]; p < 0.01) |
| E86 | 0.98 (95% CI [0.88; 1.09]; p = 0.69) |
| I63 | 1.08 (95% CI [0.99; 1.18]; p = 0.09) |
| J44 | 1.61 (95% CI [1.44; 1.79]; p < 0.01) |

Appendix 5: Rate Ratio between different Age Groups for ≥ 23°C

| **Diagnosis Code** | **Age Group 1** | **Age Group 2** | **Relative Risk Ratio (95% CI; p-value)** |
| --- | --- | --- | --- |
| All Diagnosis | 0-4 | 5-19 | 1,60 (95% CI [1,23; 2,08]; p < 0.01) |
|  | 0-4 | 20-59 | 0,84 (95% CI [0,68; 1,05]; p < 0.01) |
|  | 0-4 | 60+ | 0,18 (95% CI [0,15; 0,21]; p < 0.01) |
|  | 5-19 | 60+ | 0,11 (95% CI [0,09; 0,14]; p < 0.01) |
|  | 20-59 | 5-19 | 1,90 (95% CI [1,47; 2,45]; p < 0.01) |
|  | 20-59 | 60+ | 0,21 (95% CI [0,18; 0,25]; p < 0.01) |
| E86 | 0-4 | 5-19 | 1,96 (95% CI [1,47; 2,61]; p < 0.01) |
|  | 0-4 | 20-59 | 7,72 (95% CI [4,73; 12,62]; p < 0.01) |
|  | 0-4 | 60+ | 1,05 (95% CI [0,82; 1,33]; p < 0.01) |
|  | 5-19 | 60+ | 0,53 (95% CI [0,40; 0,71]; p < 0.01) |
|  | 20-59 | 5-19 | 0,25 (95% CI [0,15; 0,43]; p < 0.01) |
|  | 20-59 | 60+ | 0,14 (95% CI [0,08; 0,22]; p < 0.01) |
| I63 | 0-4 | 5-19 | 0,20 (95% CI [0,02; 1,71]; p < 0.01) |
|  | 0-4 | 20-59 | 0,01 (95% CI [0,00; 0,08]; p < 0.01) |
|  | 0-4 | 60+ | <0,01 (95% CI [0,00; 0,02]; p < 0.01) |
|  | 5-19 | 60+ | 0,01 (95% CI [0,01; 0,03]; p < 0.01) |
|  | 20-59 | 5-19 | 17,00 (95% CI [6,90; 41,89]; p < 0.01) |
|  | 20-59 | 60+ | 0,23 (95% CI [0,18; 0,29]; p < 0.01) |
| J44 | 0-4 | 5-19 | 0,50 (95% CI [0,13; 2,00]; p < 0.01) |
|  | 0-4 | 20-59 | 0,06 (95% CI [0,02; 0,18]; p < 0.01) |
|  | 0-4 | 60+ | 9,00 (95% CI [3,87; 20,92]; p < 0.01) |
|  | 5-19 | 60+ | 0,01 (95% CI [0,00; 0,04]; p < 0.01) |
|  | 20-59 | 5-19 | 0,02 (95% CI [0,01; 0,05]; p < 0.01) |
|  | 20-59 | 60+ | 0,21 (95% CI [0,16; 0,28]; p < 0.01) |
